# GV1001, an hTERT-Derived Peptide, Prevents Cisplatin-Induced Nephrotoxicity by Preserving Mitochondrial Function

**DOI:** 10.3390/cells14221818

**Published:** 2025-11-19

**Authors:** Wei Chen, Cheyenne Beheshtian, Seojin Kim, Reuben Kim, Sangjae Kim, No-Hee Park

**Affiliations:** 1 The Shapiro Family Laboratory of Viral Oncology and Aging Research, UCLA School of Dentistry, 714 Tiverton Ave, Los Angeles, CA 90095, USA; wchen@dentistry.ucla.edu (W.C.); cbeheshtian@dentistry.ucla.edu (C.B.); sj25kim@gmail.com (S.K.); rkim@dentistry.ucla.edu (R.K.); 2 UCLA Jonsson Comprehensive Cancer Center, 10833 Le Conte Ave, Los Angeles, CA 90095, USA; 3 Teloid Inc., 3580 Wilshire Blvd, Los Angeles, CA 90024, USA; 4 Department of Medicine, David Geffen School of Medicine at UCLA, 10833 Le Conte Ave, Los Angeles, CA 90095, USA

**Keywords:** GV1001, cisplatin, nephrotoxicity, mitochondria, reactive oxygen species

## Abstract

**Highlights:**

**What are the main findings?**

- GV1001 prevents cisplatin-induced nephrotoxicity in mice, as evidenced by the reversal of cisplatin-induced histopathological abnormalities, inflammatory responses, apoptotic cell death, and elevations in serum and renal injury markers.
- GV1001 preserves mitochondrial integrity and function against cisplatin-induced damages.

**What is the implication of the main finding?**

- GV1001 can serve as a novel protective agent against cisplatin-induced nephrotoxicity.

**Abstract:**

GV1001, a multifunctional peptide, has shown numerous biomedical activities, including antioxidant, anti-inflammatory, anti-Alzheimer’s, and anti-atherosclerotic effects, and protects mitochondria from cytotoxic agents. Cisplatin is a widely used chemotherapeutic agent against cancers, but its clinical utility is limited by nephrotoxicity driven by mitochondrial dysfunction in renal epithelial cells. Here, we investigated whether GV1001 protected against cisplatin-induced nephrotoxicity (CIN) in vivo and preserved mitochondrial integrity in human renal epithelial cells in vitro. In mice, GV1001 substantially mitigated CIN by significantly reducing histological damage, kidney injury marker expression, macrophage infiltration, endothelial-to-mesenchymal transition, inflammation, and apoptosis. In cultured renal epithelial cells, GV1001 maintained mitochondrial membrane potential, preserved ATP production, and prevented mitochondrial membrane peroxidation possibly by binding to cardiolipin. GV1001 also reduced the level of reactive oxygen species (ROS), suppressed cytochrome c release into the cytosol, and inhibited activation of apoptosis-related pathways elicited by cisplatin. Collectively, these findings demonstrated that GV1001 might protect kidney from cisplatin through maintaining mitochondrial structure and function and suppressing downstream injury cascades in renal epithelial cells. By directly targeting the mitochondrial mechanisms underlying cisplatin toxicity, GV1001 represents as a promising therapeutic strategy to mitigate CIN and improve the safety of cisplatin-based chemotherapy.

## 1. Introduction

Cisplatin is a chemotherapeutic agent that is widely used to treat solid tumors such as prostate, head and neck, ovarian, and lung cancers [1]. However, its use is often limited by nephrotoxicity, which can progress to renal failure [2,3]. Because the kidney eliminates cisplatin through glomerular filtration and tubular secretion [4], the drug accumulates preferentially in the S3 segment of proximal tubules [5]. Although hydration therapy is routinely applied, its protective efficacy remains limited [6], underscoring the need for new strategies to prevent cisplatin-induced kidney injury. In addition to inducing nuclear DNA damage, cisplatin also targets mitochondria [7]. Given the kidney’s high energy demand for tubular reabsorption and systemic homeostasis [8], mitochondrial dysfunction plays a central role in nephrotoxicity. Cisplatin-compromised mitochondria generate excessive reactive oxygen species (ROS), overwhelm cellular antioxidant defense system, and exacerbate oxidative injury [9]. Therefore, therapeutic strategies aimed at preserving mitochondrial integrity may effectively alleviate cisplatin-induced renal damage [10].

GV1001, a 16-amino acid peptide derived from human telomerase reverse transcriptase (hTERT) [11,12], exhibits broad cytoprotective activities against diverse cytotoxic insults by preserving mitochondrial function. This preservation results in decreased mitochondrial ROS generation [13], attenuation of vascular inflammation [14,15], and prevention of mitochondrial damage [15]. In addition, GV1001 has shown therapeutic potential in cancer, viral infections, and inflammatory diseases [16,17,18,19,20]. We previously reported that GV1001 alleviated doxorubicin-induced atherosclerosis by preventing mitochondrial injury [15]. These findings suggest that GV1001 may also protect the kidney from cisplatin-induced nephrotoxicity (CIN) through its mitochondria-preserving effects. Accordingly, in this study, we investigated whether GV1001 protected against CIN and evaluated its potential as a mitochondria-targeted therapeutic agent for preventing chemotherapy-associated kidney injury.

## 2. Materials and Methods

### 2.1. Cell Culture

Human kidney epithelial cells (HK-2; ATCC, #CRL-2190, Manassas, VA, USA) were cultured in RPMI-1640 medium (Thermo Fisher Scientific, #11875093, Waltham, MA, USA) supplemented with 10% fetal bovine serum (FBS) (Thermo Fisher Scientific, #10437028, Waltham, MA, USA). Normal human renal epithelial cells (NHREC; Lifeline Cell Technology, #FC-0012, Frederick, MD, USA) were maintained in RenaLife™ Epithelial Medium (Lifeline Cell Technology, #LM-0010, Frederick, MD, USA). The culture medium was replaced every two days, and cells were incubated at 37 °C in a humidified atmosphere containing 5% CO_2_.

### 2.2. Animal Experiments and Animal Welfare

Thirty-two 8-week-old male mice (C57BL/6J background; Jackson Laboratory, #000664, Bar Harbor, ME, USA) were purchased and acclimated for one week under quarantine conditions. Mice were housed in groups with free access to standard chow and water, as described previously [14,18]. After acclimation for 7 days, animals were housed in the vivarium of the Division of Laboratory Animal Medicine of the University of California, Los Angeles (UCLA) and randomly assigned to four groups (*n* = 8 per group; 4 mice in a cage) as follows:

- Control Group: Received phosphate-buffered saline (PBS) via intraperitoneal injection (i.p. inj.) three times per week for 6 weeks.
- GV1001 Group: Received GV1001 (2 mg/kg) i.p. inj. three times per week for 6 weeks. This dosage has been shown to effectively suppress inflammation without inducing toxicity in mice [14].
- Cisplatin + PBS Group: Received cisplatin (2.5 mg/kg) i.p. inj. twice per week for 6 weeks, along with PBS i.p. inj. three times per week for 6 weeks. This dosage was reported to demonstrate anti-cancer activity without mortality in mice, like the dose for cisplatin treatment in cancer patients [21].
- Cisplatin + GV1001 Group: Received cisplatin (2.5 mg/kg) i.p. inj. twice per week for 6 weeks, in combination with GV1001 (2 mg/kg) i.p. inj. three times per week for 6 weeks.

Mouse health and behavior were monitored daily throughout the experimental period, and body weights were recorded weekly. At the end of the 6-week treatment regimen, mice were humanely euthanized. Mice were anesthesized with a mixture of ketamine (100 mg/kg) and xylazine (5 mg/kg) prior to euthanasia to minimize discomfort and stress. All experimental procedures were performed in strict accordance with institutional guidelines and the regulations outlined by the U.S. Department of Agriculture (USDA) Animal Welfare Act and the Public Health Service (PHS) Policy on Humane Care and Use of Laboratory Animals. All protocols were approved by the Animal Research Committee (ARC) of the UCLA under the protocol number of ARC 2019-057. GV1001 was obtained from GemVax/Kael, Inc. (Seongnam-si, Republic of Korea). Cisplatin was purchased from Selleck Chemicals LLC (#S1166, Houston, TX, USA).

### 2.3. Tissue Collection and Determination of Kidney Damages

Under general anesthesia, whole blood was collected via cardiac puncture, and both kidneys were excised from each mouse at the experimental endpoint following 6 weeks of treatments. One kidney was immediately stored at −80 °C for RNA extraction and subsequent analysis of gene expression of Kidney Injury Molecule-1(KIM-1) and Neutrophil Gelatinase-Associated Lipocalin (NGAL). The contralateral kidney was fixed in 4% paraformaldehyde for hematoxylin and eosin (H&E) and immunofluorescent staining. Collected blood was centrifuged at 2000× *g* for 15 min to obtain serum for the determination of pro-inflammatory cytokines and other renal pathogenic indicators. Serum uric acid was measured using a Uric Acid Assay kit (Cayman Chemical Co., #700320, Ann Arbor, MI, USA). BUN levels were determined using the QuantiChrom™ Urea Assay kit (BioAssay Systems LLC, #DIUR-100, Hayward, CA, USA). Serum creatinine concentrations were quantified using a Creatinine Colorimetric Assay kit (Cayman Chemical Co., #700460, Ann Arbor, MI, USA), following the manufacturer’s instructions.

Tubular injury was evaluated on hematoxylin and eosin (H&E)–stained kidney sections by quantifying the percentage of tubules exhibiting epithelial cell necrosis, cast formation, or tubular dilatation. At least 10 randomly selected cortical fields per sample were analyzed under high-power magnification as previously described [22,23]. Tubular injury was graded based on the extent and severity of histopathological changes as follows: Grade 0: Normal tubules without detectable epithelial cell necrosis, luminal debris, or dilatation; Grade 1: Mild injury affecting <25% of tubules, characterized by focal epithelial cell swelling, occasional necrotic cells, or minimal cast formation; Grade 2: moderate injury involving 25–49% of tubules, showing evident epithelial cell necrosis, luminal cast accumulation, and tubular dilation; Grade 3: Severe injury involving 50–74% of tubules, with extensive epithelial necrosis, denudation of the tubular basement membrane, and widespread cast formation; Grade 4: Diffuse injury affecting ≥75% of tubules, characterized by near-complete tubular necrosis, luminal obstruction with dense casts, and marked tubular dilatation.

### 2.4. Immunofluorescence (IF) Staining

Kidney paraffin sections and fixed human renal epithelial cells were incubated with primary antibodies or specific probes, including F4/80 (Abcam, #ab6640, Cambridge, UK), α-SMA (alpha smooth muscle actin, MilliporeSigma, #A2547, Burlington, MA, USA), TNF-α (Abcam, #ab6671, Cambridge, UK), KIM-1 (Cell Signaling Technology, #CST14971, Danvers, MA, USA), E-Cadherin (BD Biosciences, #610181, San Jose, CA, USA), PARP (Cell Signaling Technology, #CST9542, Danvers, MA, USA), Cleaved Caspase 3 (Cell Signaling Technology, #CST9661, Danvers, MA, USA), p-p38 (Cell Signaling Technology, #CST9211, Danvers, MA, USA), p38 (Cell Signaling Technology, #CST9212, Danvers, MA, USA), COX-4 (Cell Signaling Technology, #CST4850, Danvers, MA, USA), NDUFS1 (Cell Signaling Technology, #CST70264, Danvers, MA, USA), SDHB (Cell Signaling Technology, #CST92649, Danvers, MA, USA), UQCRFS1(Cell Signaling Technology, #CST95231, Danvers, MA, USA), Cytochrome C (Invitrogen, #MA5-11674, Carlsbad, CA, USA), IL-6 (Thermo Fisher Scientific, #701028, Waltham, MA, USA), p65 (Santa Cruz Biotechnology, #SC-8008, Dallas, TX, USA), Bax (Santa Cruz Biotechnology, #SC-20067, Dallas, TX, USA), p53 (Santa Cruz Biotechnology, #SC-126, Dallas, TX, USA), IL-1β (Santa Cruz Biotechnology, #SC-32294, Dallas, TX, USA), γ-H2AX (MilliporeSigma, #05-636, Burlington, MA, USA), p-p65 (Cell Signaling, #CST-3036, Danvers, MA, USA), OCT-2 (Thermo Fisher Scientific, #PA580015, Waltham, MA, USA), Cisplatin modified DNA antibody (Abcam, #ab103261, Cambridge, UK), or GV1001 polyclonal antibody (GemVax/Kael, Inc., Seongnam-si, Republic of Korea).

Mitochondria-or ROS-specific probes included MitoSox^TM^ Red Mitochondrial Superoxide Indicator (Invitrogen, #M36008, Carlsbad, CA, USA), Fluorescent Intracellular ROS probe (MilliporeSigma, #MAK143, Burlington, MA, USA), MitoTracker Red CMXRos (Cell Signaling Technology, #CST9082, Danvers, MA, USA), Biotracker^TM^Mitochondrial FerroGreen live cell probe (Mito-FerroGreen) (EMD Millipore Corp., #SCT262, Burlington, MA, USA), Mitochondrial Lipid Peroxide Live Cell Ferroptosis Indicator (EMD Millipore Corp., #SCT261, Burlington, MA, USA), NAO (Acridine Orange 10-nonyl bromide, MilliporeSigma, #A7847, Burlington, MA, USA).

After incubation with Alexa Fluor 488- or 594-conjugated secondary antibodies (Thermo Fisher Scientific, Waltham, MA, USA) for 1 h at room temperature, slides or fixed cells were mounted using VECTASHIELD^®^ Antifade Mounting Medium with DAPI (Vector Laboratories, #H1200, Burlingame, CA, USA). Images were captured with a confocal fluorescence microscope (Carl Zeiss, LSM 700, Oberkochen, Germany).

All experiments were conducted according to the manufacturers’ instructions and previously published protocols [15,24]. Images were analyzed using ImageJ (v1.54). Background subtraction was performed with a rolling-ball radius of 50 pixels. Thresholding was applied using Otsu’s method [25] and identical thresholds were used for all images within each experiment. Mean fluorescence intensity was normalized to the average DAPI intensity within the same field. All quantifications were performed blinded to experimental conditions.

### 2.5. Apoptosis Assessment

Apoptotic cells from human renal epithelial cell cultures or mouse renal tissues were detected using a Terminal Deoxynucleotidyl Transferase dUTP Nick-End Labeling (TUNEL) assay kit (Cell Signaling, #25879, Danvers, MA, USA) according to the manufacturer’s protocols.

### 2.6. Quantitative Real-Time Polymerase Chain Reaction (RT-qPCR)

We extracted total RNA from human renal epithelial cells and mouse kidney tissues using the RNeasy Micro Kit (Qiagen, #74004, Valencia, CA, USA). Complementary DNA (cDNA) was synthesized from total RNA using the SuperScript™ III First-Strand Synthesis Kit (Thermo Fisher Scientific, #18080044, Waltham, MA, USA) under the following conditions: 65 °C for 5 min, 25 °C for 2 min, and 45 °C for 60 min. Quantitative PCR was performed with the PowerUp™ SYBR™ Green Master Mix (Thermo Fisher Scientific, #A25742, Waltham, MA, USA) according to the manufacturer’s instructions. Glyceraldehyde-3-phosphate dehydrogenase (GAPDH) was used as the internal reference gene. Relative transcript levels were calculated using the comparative ΔCq method and expressed as fold changes (2^−ΔΔCq^). Primer sequences used for RT-qPCR were listed in Table S1 (Supplemental Materials).

### 2.7. Western Blotting

Western blotting was performed using the whole-cell extracts (WCEs) of the cultured human renal epithelial cells as described elsewhere [26].

### 2.8. Enzyme-Linked Immunosorbent Assay (ELISA)

To detect the levels of Interleukin-6 (IL-6), Interleukin-1β (IL-1β), and Tumor necrosis factor-alpha (TNF-α) in mouse serum, commercially available enzyme-linked immunosorbent assay kits (Invitrogen, Carlsbad, CA, USA) were used according to the manufacturer’s instructions as described previously [27].

### 2.9. ATP Detection Assay

Human renal epithelial cells were seeded into a 6-well plate at a density of 2 × 10^5^ cells/well in 2 mL of DMEM/F12 medium. After cells were treated with GV1001 (10 μg/mL), cisplatin (25 μM) alone or in combination with GV1001 for 24 h, intracellular ATP content was measured using the ATP Detection Assay Kit (#700410, Cayman Chemical, Ann Arbor, MI, USA), following the manufacturer’s instructions as described previously by others [28,29]. Briefly, after washing the cells with pre-chilled PBS, 1 mL of 1× ATP Detection Sample Buffer was added to each well. These cells were homogenized by pipetting the buffer up and down several times, and the cell lysates were then transferred to pre-chilled tubes. The Reaction Mixture was prepared by mixing D-Luciferin and Luciferase with 1× ATP Detection Assay Buffer. One hundred microliters of the freshly prepared Reaction Mixture and 10 μL of cell lysates were added to a white opaque 96-well plate in triplicates. The plate was covered and incubated at room temperature for 20 min. The ATP concentration was determined by measuring luminescence using the spectrometer (Bio-Rad Laboratories, #PR4100, Hercules, CA, USA).

### 2.10. Mitochondrial Membrane Potential (MMP) Detection Assay

Human renal epithelial cells were plated onto a chamber slide (Lab-Tek II, Nunc, C6932, Rochester, NY, USA). and incubated in an incubator set for 24 h at 37 °C and equilibrated with 95% air and 5% CO_2_. After cells were treated with GV1001, cisplatin alone or in combination with GV1001 for another 24 h, The working solution of MT-1 probe (Fish scientific, # NC1933275, Waltham MA, USA) were added into the cell culture medium. These cells were incubated for 30 min at 37 °C, and then washed with HBSS twice. After fixation cells with 4% paraformaldehyde for 20 min, the cells were observed under the confocal fluorescence microscope (Carl Zeiss, LSM 700, Oberkochen, Germany) at an excitation wavelength of 530–560 nm and an emission wavelength of 570–640 nm.

### 2.11. Labeling GV1001 with Fluorescein Isothiocyanate (FITC)

GV1001 peptide was dissolved in 0.1 M sodium bicarbonate buffer (MilliporeSigma, #S6014, Burlington, MA, USA), pH 9.0–9.5, to a final concentration of 2 mg/mL. Fluorescein isothiocyanate (FITC) (MilliporeSigma, #F3651, Burlington, MA, USA) was dissolved in anhydrous dimethyl sulfoxide (DMSO) to the concentration of 1 mg/mL. The FITC stock solution was then added to the GV1001 solution at 5:1 molar ratio (FITC: GV1001). The mixture was gently stirred in the dark at 4 °C for 2–8 h, after which NH_4_Cl solution (50 mM) was added to quench excess FITC. The reaction was incubated in the dark for another 2 h at 4 °C. The mixture was passed through a G-25 Sephadex column equilibrated with PBS to separate free FITC from FITC-labeled GV1001 [30].

### 2.12. Lipid Binding Assay

An ELISA-based lipid binding assay was performed using 96-well white opaque tissue culture plates (Corning Life Science, #353296, Union City, CA, USA) coated with 2.5 µg/well of either cardiolipin (MilliporeSigma, #C1649, Rockville, MD, USA) or phosphatidylinositol 4,5-bisphosphate (PI(4,5)P_2_; Echelon Biosciences, #P-4508, Salt Lake City, UT, USA) as a negative control lipid. Plates were coated overnight at 4 °C in the dark and subsequently blocked for 1 h with assay buffer (10 mM Bis-Tris and 10 mM CaCl_2_) containing 0.5% skim milk and 2% fatty acid–free BSA, as previously described [31]. Serial dilutions of FITC-labeled GV1001 were then added (50 µL/well, in quadruplicate) and incubated for 2 h at 37 °C to allow lipid–peptide interaction. After washing the plate five times with wash buffer (10 mM HEPES plus 100 mM NaCl) to remove unbound GV1001, fluorescence intensity was measured at an excitation wavelength of 490 nm and emission wavelength of 520 nm using a spectrophotometer (Bio-Rad Laboratories, #PR4100, Hercules, CA). Negative control included wells without lipid coating, wells without GV1001 incubation, and wells coated with PI(4,5)P_2_ to verify the specificity of GV1001 binding to cardiolipin. The relative amount of GV1001 bound to each lipid was calculated using the formula: Relative Binding (%) = (F−F_blank_)/(Fmax − F_blank_) × 100, where F represented the measured fluorescence intensity. The results were expressed as percentage binding and fitted to a sigmoidal dose–response curve to confirm specific, saturable interaction between GV1001 and cardiolipin.

### 2.13. Isolation of Mitochondria and Protein Analysis from the Mitochondria and Cytosol

Cellular mitochondria were isolated using a Mitochondrial Isolation kit for Mammalian Cells (Thermo Fisher Scientific, #89874, Waltham, MA, USA) according to the manufacturer’s instructions. Cells treated with cisplatin, alone or in combination with GV1001, were washed twice with PBS and harvested. The collected cells were resuspended in a fractionation buffer and incubated on ice for 5 min. Samples were then centrifuged at 12,000× *g* for 15 min to separate the cytosolic fraction (supernatant) from the mitochondrial fraction (pellet). Proteins from both mitochondria and mitochondria-free cytosol were subsequently analyzed by Western blotting.

### 2.14. Statistical Analyses

All statistical analyses were performed using GraphPad Prism 9 (GraphPad Software, Boston, MA, USA). For multiple group comparisons, the Kruskal–Wallis test followed by Dunn’s post hoc analysis was used. A *p*-value < 0.05 was considered statistically significant. All in vitro experiments were independently repeated at least five times, and data were presented as the mean ± SE.

## 3. Results

### 3.1. Effect of GV1001 on Cisplatin-Induced Nephrotoxicity

**Figure 1:**
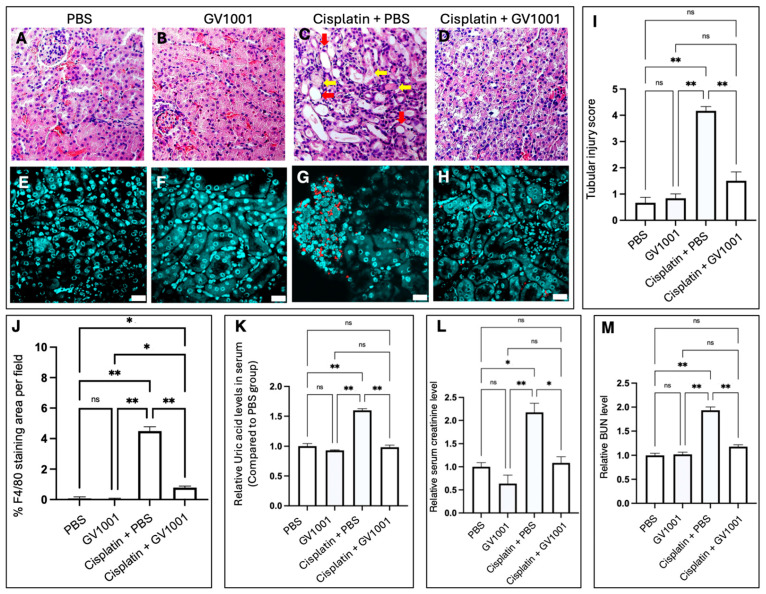
Representative H&E-stained kidney sections from mice treated with PBS (**A**), GV1001 (**B**), cisplatin + PBS (**C**), or cisplatin + GV1001 (**D**). Red arrows indicated dilated tubules with epithelial cell flattening and loss; yellow arrows highlighted regions of tubular necrosis and proteinaceous casts. Images of H&E staining were shown at 200× magnifications. Representative immunofluorescence images showing monocytes/macrophages stained with F4/80 antibody (red) in renal epithelial tissue from mice treated with PBS (**E**), GV1001 (**F**), cisplatin + PBS (**G**), or cisplatin + GV1001 (**H**). Nuclei were counterstained with DAPI (blue). Scale bar for immunofluorescence images: 20 μm. (**I**). Quantification of tubular injury score from H&E-stained kidney sections. (**J**). Quantification of F4/80-positive staining area per field using ImageJ software. (**K**–**M**). Serum levels of uric acid, creatinine, and BUN in each treatment group. Data were analyzed by one-way ANOVA. ns, not significantly different; * *p* < 0.05; ** *p* < 0.01 with *n* = 8 per group. All H&E and immunofluorescence staining data were obtained from five independent replicates per mouse.

Histological analyses revealed that cisplatin treatment induced pronounced tubular cystic dilation and formation of proteinaceous casts, classic hallmarks of nephrotoxicity [32], compared with kidneys from mice treated with PBS or GV1001 alone (Figure 1A–C). Remarkably, co-treatment with GV1001 prevented cisplatin-induced pathological alterations, underscoring its strong protective effect (Figure 1D). Consistent with these observations, semi-quantitative scoring of H&E-stained sections demonstrated that GV1001 markedly attenuated cisplatin-induced tubular dilation, necrosis, and cast formation (Figure 1I).

Immunofluorescence staining further showed that cisplatin promoted monocyte/macrophage infiltration within the renal cuboidal epithelium (Figure 1E–G), whereas GV1001 co-treatment significantly reduced immune cell accumulation (Figure 1H,J). Moreover, key biomarkers and pathogenic indicators of cisplatin-induced CIN, including uric acid, creatinine, and blood urea nitrogen (BUN) from serum [33], were all markedly elevated in cisplatin-treated mice. GV1001 alone did not alter the baseline values but significantly suppressed these cisplatin-induced elevations (Figure 1K–M). In addition, TUNEL assay demonstrated that cisplatin induced apoptosis of renal tubular cells, whereas co-treatment of GV1001 effectively prevented this apoptotic response, thereby limiting kidney injury (Figure S1A,B). Collectively, these findings demonstrate that GV1001 mitigates the cisplatin-induced histological alterations, monocyte/macrophage infiltration, and apoptosis of renal epithelial cells, highlighting its potential as a protective agent against chemotherapy-associated nephrotoxicity.

### 3.2. Effect of GV1001 on the Expression of Genes Associated with the Cisplatin-Induced Kidney Injury

**Figure 2:**
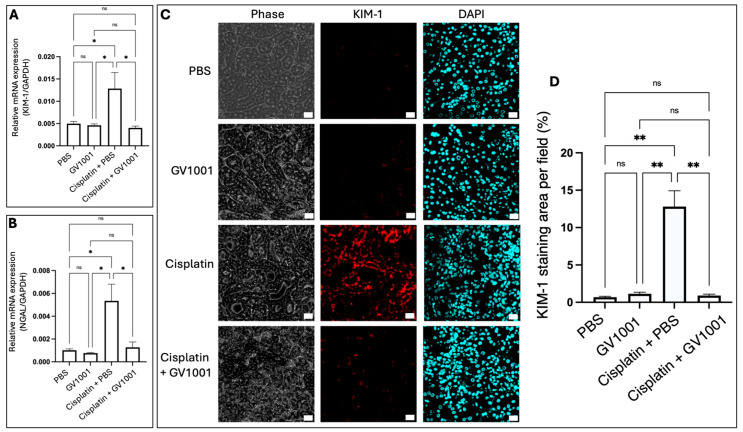
(**A**,**B**). RT-qPCR analysis of KIM-1 and NGAL mRNA expression in renal tissues of mice treated with PBS, GV1001, Cisplatin + PBS, or Cisplatin + GV1001. (**C**). Representative immunofluorescence images showing KIM-1 (red) expression in the renal cuboidal epithelium. Nuclei were counterstained with DAPI (blue). Scale bar: 20 μm. (**D**). Quantification of KIM-1 positive staining per field using ImageJ software. Data were presented as mean ± SEM (*n* = 8 per group). Data were analyzed by one-way ANOVA. ns = not significantly different; * *p* < 0.05; ** *p* < 0.01. All RT-qPCR experiments were performed in quintuplicate. Immunofluorescence staining data were obtained from five independent replicates per mouse.

As shown in Figure 2A,B, the RT-qPCR analysis revealed that cisplatin markedly upregulated the mRNA expression of both KIM-1 and NGAL, two established biomarkers of CIN [34], in renal tissues. Co-treatment with GV1001 significantly suppressed the cisplatin-induced overexpression of KIM-1 and NGAL. Consistently, immunofluorescence staining revealed that cisplatin strongly induced KIM-1 expression in renal epithelium (Figure 2C,D), whereas GV1001 co-treatment markedly attenuated this induction.

### 3.3. Effect of GV1001 on the Cisplatin-Induced Epithelial-to-Mesenchymal Transition (EMT) of Mouse Kidney Epithelial Cells

**Figure 3:**
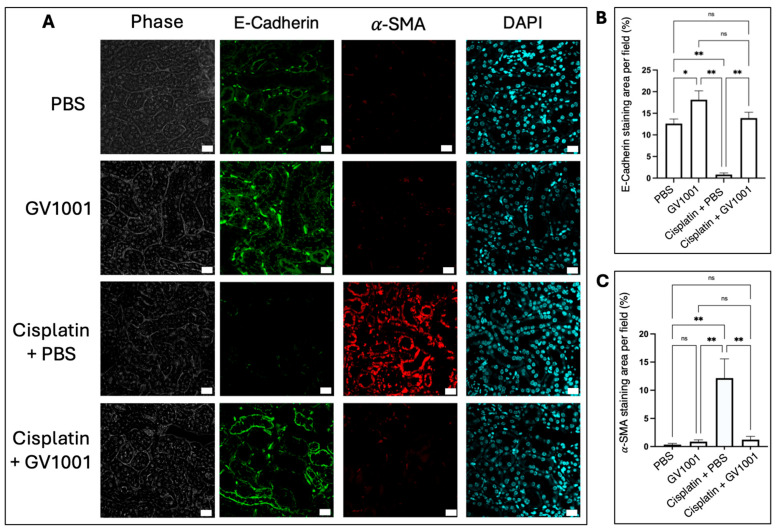
(**A**). Representative immunofluorescence images of E-cadherin (green) and α-SMA (red) in the renal cuboidal epithelium of mice treated with PBS, GV1001, cisplatin or cisplatin + GV1001. Nuclei were counterstained with DAPI (blue). Scale bar: 20 μm. (**B**,**C**). Quantification of E-cadherin and α-SMA-positive staining areas per field using ImageJ software. Data were presented as mean ± SEM (*n* = 8 per group) and were analyzed by one-way ANOVA. ns, not significantly different; * *p* < 0.05; ** *p* < 0.01. All experiments were performed in quintuplicate.

To further evaluate the protective role of GV1001 against cisplatin-induced CIN, we examined whether GV1001 inhibited the cisplatin-induced EMT of kidney cuboidal epithelial cells. As shown in Figure 3A–C, cisplatin treatment induced EMT, characterized by marked downregulation of E-Cadherin (an epithelial cell marker) and a concomitant upregulation of α-SMA (a key indicator of mesenchymal cells) in the renal proximal epithelium. Co-administration of GV1001 with cisplatin preserved epithelial integrity and suppressed mesenchymal transition in renal epithelium.

### 3.4. Effect of GV1001 on the Cisplatin-Induced Systemic and Renal Inflammation in Mice

**Figure 4:**
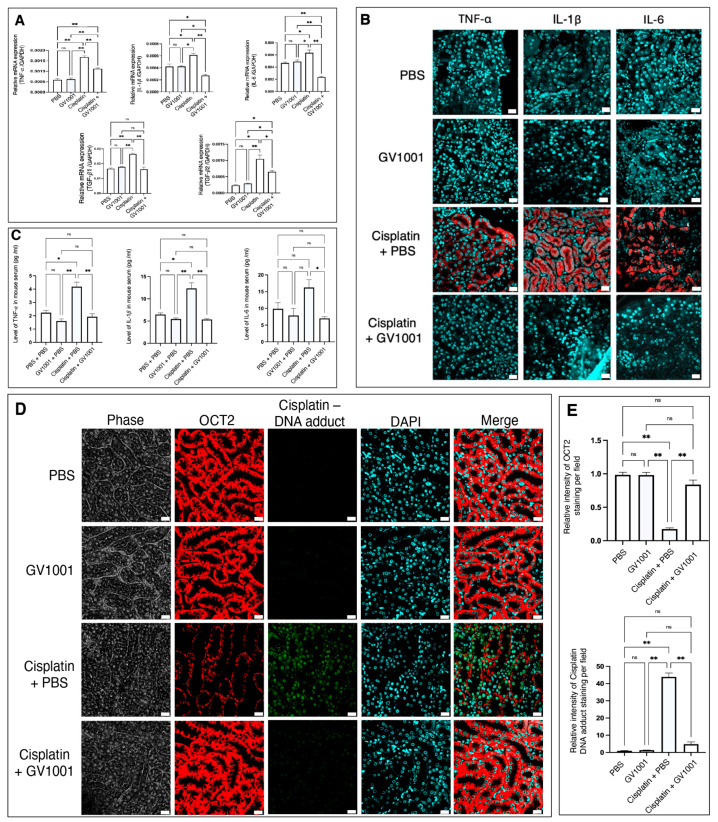
(**A**). RT-qPCR analysis showing that GV1001 suppressed cisplatin-induced upregulation of TNF-α, IL-1β, IL-6, TGF-β1, and TGF-β2 mRNA expression in renal tissues. GAPDH was used as an internal control. (**B**). Representative immunofluorescence images of TNF-α, IL-1β, and IL-6 in the renal tubular epithelium of mice treated with PBS, GV1001, cisplatin, or cisplatin + GV1001. Cytokines were shown in red, and nuclei were counterstained with DAPI (blue). Scale bar: 20 μm. (**C**). ELISA analysis of serum TNF-α, IL-1β, and IL-6 levels in each treatment group. (**D**). Representative immunofluorescence images showing OCT2 expression and cisplatin-DNA adduct accumulation in the renal tubular epithelium. OCT2 was shown in red, cisplatin-DNA adduct was shown in green, nuclei were counterstained with DAPI (blue). Scale bar: 20 μm. (**E**). Quantification of OCT2-positive and cisplatin–DNA adduct–positive staining areas per field using ImageJ software. Data were presented as mean ± SEM (*n* = 8 per group) and analyzed by one-way ANOVA. Symbols indicated ns, not significantly different; * *p* < 0.05; ** *p* < 0.01. All experiments were performed in quintuplicate.

We next evaluated the effects of GV1001 on cisplatin-induced inflammation in both kidney tissue and blood serum. As shown in Figure 4A, cisplatin treatment markedly increased the mRNA expression of pro-inflammatory cytokines, including TNF-α, IL-1β, IL-6, TGF-β1 and TGF-β2 in renal tissues, whereas co-treatment with GV1001 significantly inhibited the cisplatin-induced elevations. Consistently, immunofluorescence staining revealed enhanced intraepithelial expression of these cytokines, following cisplatin exposure, which was markedly attenuated by GV1001 co-treatment (Figure 4B). Furthermore, ELISA analysis demonstrated that cisplatin administration significantly elevated serum levels of TNF-α, IL-1β, and IL-6, while GV1001 effectively reduced these increases (Figure 4C). In addition, as shown in Figure 4D,E, cisplatin exposure decreased the expression of Organic Cation Transporter 2 (OCT2) in the basolateral membrane of proximal tubular cells, whereas GV1001 restored OCT2 expression. Moreover, cisplatin induced substantial cisplatin–DNA adduct formation in renal cells, but GV1001 co-treatment markedly prevented cisplatin–DNA adduct accumulation, suggesting protection against cisplatin-induced genotoxic stress.

### 3.5. Effects of Cisplatin and GV1001 on Mitochondrial Mass and Function

**Figure 5:**
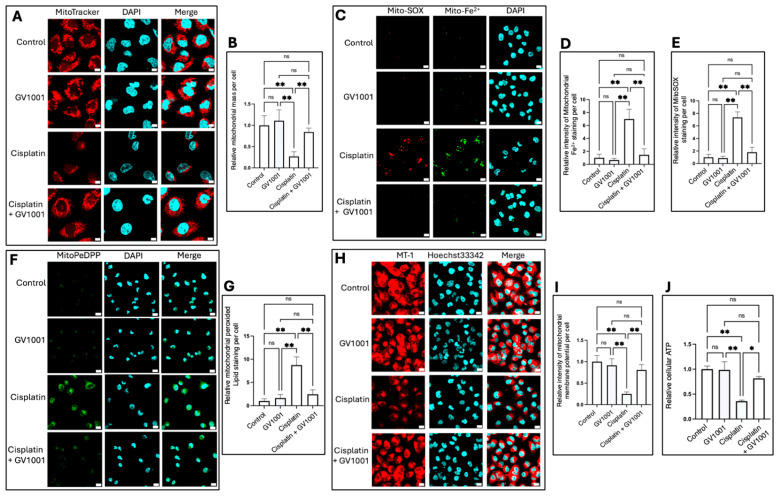
(**A**). Representative immunofluorescence images of mitochondrial mass detected using MitoTracker™ Red CMXRos (red) in HK-2 cells treated with cisplatin, GV1001, or cisplatin + GV1001 for 24 h. Scale bar: 10 µm. (**B**). Quantification of mitochondrial mass using *ImageJ* analysis. (**C**,**F**,**H**). Representative immunofluorescence images showing mitochondrial ROS and mitochondrial ferrous iron (Mito-Fe^2+^) (**C**), lipid peroxidation (MitoPeDPP staining) (**F**), and MT-1 red fluorescence (indicator of MMP) (**H**). Scale bar: 20 μm. (**D**,**E**,**G**,**I**). Quantification of mitochondrial ROS, mitochondrial Fe^2+^, lipid peroxidation, and MMP levels using ImageJ software. (**J**). Relative cellular ATP levels in HK-2 cells under each treatment condition. Data were presented as mean ± SEM and analyzed by one-way ANOVA. ns: not significantly different; * *p* < 0.05; ** *p* < 0.01. All experiments were performed in quintuplicate.

To elucidate the protective mechanisms of GV1001 against CIN, we examined its effects on mitochondrial structure and function in renal epithelial cells. As shown in Figure 5A,B, cisplatin markedly decreased mitochondrial mass in HK-2 cells, whereas GV1001 co-treatment effectively prevented these structural impairments. Consistent with this observation, cisplatin significantly increased both mitochondrial reactive oxygen species (ROS) (Figure 5C,E) and cytosolic ROS levels (Figure S2), which were markedly suppressed by GV1001.

Moreover, cisplatin elevated mitochondrial ferrous iron (Fe^2+^) accumulation (Figure 5C,D) and lipid peroxidation (Figure 5F,G), both of which were substantially reduced by GV1001. Cisplatin also significantly diminished MT-1 red fluorescence intensity, indicating mitochondrial membrane potential (MMP) depolarization, while GV1001 restored MMP to near-control levels (Figure 5H,I). In line with these findings, cisplatin markedly suppressed cellular ATP production, whereas GV1001 co-treatment restored ATP levels in HK-2 cells (Figure 5J).

Similar protective effects were observed in normal human renal epithelial cells (NHRECs). As shown in Figure S3A,B, cisplatin markedly decreased MMP in NHRECs, which was restored by GV1001. Likewise, cisplatin-induced mitochondrial ROS accumulation (Figure S3C,D) and cisplatin-reduced mitochondrial mass (Figure S3E,F) were both reversed by GV1001 co-treatment.

To further determine whether cisplatin affected the electron transport chain (ETC), we analyzed the expression of mitochondrial ETC complexes I–IV using immunofluorescence staining. Specifically, we assessed complex I (NADH dehydrogenase (ubiquinone) Fe-S protein 1, NDUFS1), complex II (succinate dehydrogenase complex iron–sulfur subunit B, SDHB), complex III (ubiquinol–cytochrome c reductase Rieske iron–sulfur polypeptide 1, UQCRFS1), and complex IV (cytochrome c oxidase subunit IV, COX-4). As shown in Figures S4 and S5, cisplatin markedly reduced the levels of NDUFS1, SDHB, UQCRFS1, and COX-4 in HK-2 and NHRECs. Notably, GV1001 co-treatment restored all four ETC complexes to near control levels, indicating that GV1001 preserved mitochondrial ETC integrity under cisplatin-induced stress. Collectively, these findings demonstrate that GV1001 protects renal epithelial cells from cisplatin-induced injury, at least in part, by maintaining mitochondrial mass, membrane potential, and electron transport chain function.

### 3.6. Effect of GV1001 on Cisplatin-Caused Apoptosis in Human Renal Epithelial Cells

**Figure 6:**
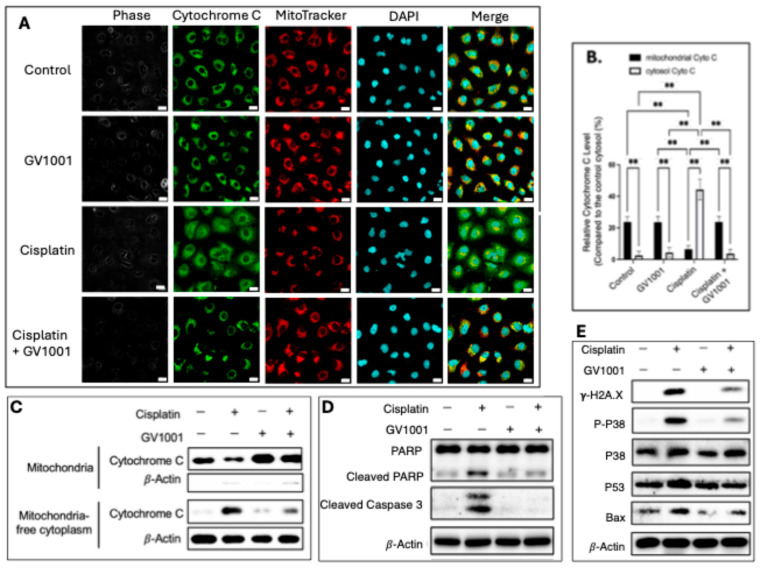
(**A**). Representative immunofluorescence images of cytochrome c (green) in HK-2 cells treated with GV1001, cisplatin, or cisplatin + GV1001. Mitochondria were stained with MitoTracker™ (red), and nuclei were counterstained with DAPI (blue). Scale bar: 20 µm. (**B**). Quantification of cells exhibiting cytosolic cytochrome c localization per field. (**C**). Western blot analysis of cytochrome c in mitochondrial and cytosolic fractions following subcellular fractionation. (**D**,**E**). Western blot analyses of proteins associated with apoptosis (cleaved PARP, cleaved caspase-3, p-p38, p53, Bax) and DNA damage (γ-H2AX) in cells treated with vehicle, GV1001, cisplatin, or cisplatin + GV1001. Data were presented as mean ± SEM and analyzed by one-way ANOVA. ns, not significantly different; ** *p* < 0.01. All experiments were performed in quintuplicate.

As shown in Figure 6A,B, immunofluorescence staining revealed a pronounced release of cytochrome c from mitochondria into the cytosol in cisplatin-treated HK-2 cells, whereas GV1001 effectively prevented cytochrome c translocation. MitoTracker™ was used as a mitochondrial marker to visualize subcellular localization. Similar results were observed in NHRECs, as shown in Figure S3E,F, where cisplatin induced cytochrome c release that was blocked by GV1001 co-treatment.

Consistent with these findings, subcellular fractionation and immunoblotting confirmed that cisplatin caused cytosolic translocation of cytochrome c, while GV1001 inhibited this release (Figure 6C). As shown in Figure 6D, cisplatin treatment markedly activated caspase-3, whereas GV1001 suppressed caspase-3 activation. One of the major substrates of caspase-3, Poly (ADP-ribose) polymerase (PARP), was also examined; cisplatin significantly increased cleaved PARP accumulation, which was notably reduced by GV1001. Furthermore, cisplatin-induced DNA damage activated both the p53 and p38 signaling pathways. As shown in Figure 6E, cisplatin increased the expression of γ-H2AX (a marker of DNA double-strand breaks), phosphorylated p38 (p-p38), p53, and Bax, while these effects were substantially attenuated by GV1001 co-treatment.

In addition, TUNEL assay results (Figure S6) demonstrated that cisplatin markedly increased the number of TUNEL-positive apoptotic cells, whereas GV1001 significantly reduced cisplatin-induced apoptosis in both HK-2 and NHRECs. These results are consistent with the reduced caspase-3 activity and apoptotic marker expression observed in cells co-treated with GV1001 and cisplatin, confirming that GV1001 effectively inhibits cisplatin-induced apoptotic signaling in renal epithelial cells.

### 3.7. GV1001 Binds to Mitochondrial Cardiolipin

**Figure 7:**
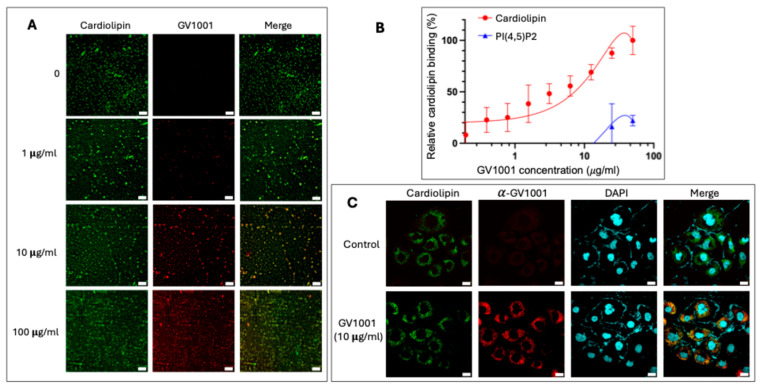
(**A**). Representative immunofluorescence images showing dose-dependent binding of GV1001 (red) to cardiolipin (green). (**B**). ELISA-based lipid binding assay demonstrating the interaction between FITC-labeled GV1001 and cardiolipin or PI(4,5)P_2_ at the indicated concentrations. (**C**). Representative immunofluorescence images of GV1001 and cardiolipin in HK-2 cells treated with GV1001 (10 µg/mL). GV1001 was shown in red, cardiolipin was labeled with 10-N-nonyl acridine orange (NAO; green), and nuclei were counterstained with DAPI (blue). Scale bar: 20 µm. All experiments were performed in quintuplicate.

Given cardiolipin’s essential role in mitochondrial structure and function, and GV1001’s observed cytoprotective effects, we hypothesized that GV1001 might directly interact with cardiolipin, a unique phospholipid predominantly localized in the inner mitochondrial membrane. Immunofluorescence staining using an anti-GV1001 antibody revealed a dose-dependent binding of GV1001 to cardiolipin in vitro (Figure 7A).

To further characterize this interaction, an ELISA-based cardiolipin binding assay was performed using cardiolipin-coated plates incubated with increasing concentrations of FITC-labeled GV1001. The assay demonstrated a concentration-dependent increase in fluorescence intensity, confirming GV1001’s binding affinity to cardiolipin (Figure 7B). As a negative control, phosphatidylinositol 4,5-bisphosphate [PI(4,5)P_2_], a key lipid component of the plasma membrane, was included. GV1001 exhibited minimal binding to PI(4,5)P_2_, though a slight increase was observed at higher GV1001 concentrations, likely reflecting nonspecific interactions (Figure 7B). In addition, 10-N-nonyl acridine orange (NAO), a fluorescent probe specific for cardiolipin, was used to visualize its mitochondrial localization. Double immunofluorescence staining with NAO and anti-GV1001 antibody showed that GV1001 diffused into the cytoplasm of renal epithelial cells and colocalized with cardiolipin (Figure 7C).

## 4. Discussion

Despite decades of research and clinical trials, no effective pharmacological strategy has been established to prevent CIN in patients undergoing chemotherapy [35]. The present study demonstrates that GV1001 protects against CIN through multiple convergent mechanisms centered on mitochondrial preservation and cellular homeostasis. Cisplatin nephrotoxicity results from the interplay of mitochondrial dysfunction, oxidative stress, inflammation, and apoptosis [36], and GV1001 interrupts this pathophysiological cascade at several critical points, thereby preventing the downstream injury processes that culminates in renal failure.

Histological analyses demonstrated that GV1001 effectively prevented tubular dilation, necrosis, and cast formation, which were hallmark features of cisplatin-induced nephrotoxicity. These morphological improvements were accompanied by marked reductions in macrophage infiltration, tubular apoptosis, and biochemical indicators of renal dysfunction, including serum uric acid, creatinine, and BUN levels. The histological and biochemical amelioration elicited by GV1001 treatment reflects a robust stabilization of epithelial integrity and function. Notably, GV1001 also mitigated the cisplatin-induced upregulation of KIM-1 and NGAL expression, two key mediators of tubular dedifferentiation and inflammation [37,38], further supporting this underlying mechanism. Rather than acting as a nonspecific antioxidant, GV1001 appears to exert protective effects that preserve renal epithelial homeostasis and function under cisplatin-induced stress.

EMT is a key event linking acute injury to chronic fibrosis [39,40,41]. The ability of GV1001 to preserve E-cadherin and reduce α-SMA expression suggested it maintained epithelial polarity and intercellular adhesion, thereby interrupting the fibrogenic signaling that drove maladaptive remodeling. This finding implies that GV1001 not only prevents acute tubular necrosis but also mitigates the risk of long-term renal fibrosis, positioning it as a potential agent for preventing the transition from acute kidney injury to chronic kidney disease.

Inflammation plays a central role in amplifying cisplatin-induced injury [42,43]. Cisplatin triggers NF-κB activation and cytokine release, leading to oxidative stress, apoptosis, and suppression of epithelial transporters. Our data revealed that cisplatin-induced cytokine elevation downregulated OCT2, the transporter responsible for cisplatin uptake and clearance [44]. GV1001 prevented this downregulation, suggesting that it stabilized epithelial cells sufficiently to maintain normal transporter expression and membrane integrity. This preservation of OCT2 likely reflected the restored homeostasis of redox and inflammatory signaling, rather than increased drug uptake, and highlighted GV1001’s capacity to sustain epithelial function even under chemotoxic stress.

Cisplatin–DNA adducts represent primary molecular lesions that trigger cell-cycle arrest and apoptosis [43]. GV1001 significantly reduced the formation of these adducts, implying that it protected the nuclear genome indirectly by restoring mitochondrial redox control. Excess mitochondrial ROS can enhance DNA platination and impair nucleotide excision repair [45]. Thus, by normalizing ROS levels and energy production, GV1001 might reduce both DNA damage formation and propagation of the apoptotic response. This finding links mitochondrial protection to nuclear genome stability, illustrating how GV1001 interrupts the crosstalk between mitochondrial stress and DNA injury.

Mitochondria play a pivotal role in renal physiology, as the kidneys require high levels of energy to support filtration and reabsorption processes [46]. Excessive mitochondrial ROS generation is known to drive the damage of mitochondrial structure and function, and disruption of the MMP [47]. MMP represents the electrochemical gradient across the inner mitochondrial membrane, which is essential for ATP synthesis [10]. Mitochondrial ATP production depends on the electron transport chain (ETC), which is composed of multi-subunit protein complexes embedded within the inner mitochondrial membrane [48]. At the organelle level, GV1001 restored mitochondrial membrane potential, enhanced ATP synthesis, and normalized respiratory complex activities. These findings suggest that GV1001 directly stabilizes mitochondrial bioenergetics rather than eliciting a secondary adaptive response.

Mitochondrial dysfunction eventually induces the release of mitochondrial cytochrome c into the cytosol, where it activates caspases and initiates apoptosis [49]. Once released, cytochrome c activates its effector caspase-3, which dismantles cellular components and drives apoptosis [50]. One major substrate of caspase-3 is PARP and PARP cleavage serves as a classic marker of apoptosis [51]. Activated p53 upregulates pro-apoptotic genes, such as Bax, which promotes mitochondrial outer membrane permeabilization and the release of cytochrome c [52]. Co-administration of GV1001 inhibited mitochondrial apoptotic signaling induced by cisplatin through preventing cytochrome c release, caspase-3 and PARP cleavage, and DNA fragmentation.

Cisplatin-induced nephrotoxicity is mediated by p53-dependent apoptosis in renal tubular cells. Cisplatin was found to induce p38 phosphorylation, which is involved in signaling pathways that can lead to cell apoptosis [53]. GV1001 attenuated cisplatin-induced activation of the p53 and p38 signaling pathways, thereby mitigating kidney injury without compromising cisplatin’s antitumor efficacy. Cisplatin treatment generally decreases the activities of major antioxidant enzymes like superoxide dismutase (SOD), catalase (CAT), glutathione peroxidase (GSH-Px), and glutathione reductase (GR) in kidney [54]. Whether GV1001 treatment restores these antioxidant enzyme activities toward normal levels needs further investigation.

The identification of cardiolipin as a potential binding target of GV1001 offered a compelling mechanistic basis for its mitochondrial protective effects. Cardiolipin is a distinctive phospholipid predominantly localized within the inner mitochondrial membrane (IMM), where it plays a crucial role in maintaining membrane architecture and integrity, thereby facilitating efficient electron transport and ATP synthesis [55,56]. This mechanistic insight distinguishes GV1001 from conventional antioxidants by elucidating its dual actions in preserving mitochondrial integrity and modulating downstream apoptotic signaling.

Overall, these results established GV1001 as a mitochondria-targeted therapeutic capable of preventing cisplatin-induced nephrotoxicity. Unlike other renoprotective agents that typically target a single pathogenic pathway [57,58,59], GV1001 demonstrated broad-spectrum protection by stabilizing mitochondrial function, reducing inflammation, suppressing EMT, and inhibiting apoptosis. Future studies should evaluate whether GV1001 selectively protects normal renal tissue without diminishing cisplatin’s antitumor efficacy, as this is essential for clinical translation. In summary, GV1001 prevented CIN by integrating mitochondrial protection with anti-inflammatory, anti-fibrotic, and anti-apoptotic mechanisms. These findings suggest GV1001 as a promising mitochondria-targeted therapeutic for mitigating chemotherapy-associated kidney injury and improving cancer treatment outcomes.

Although the mouse model provides valuable mechanistic insights into GV1001’s protective effects against cisplatin-induced nephrotoxicity, several limitations exist when translating these results to human patients. Mice differ from humans in renal physiology, drug metabolism, and immune responses, which may influence both cisplatin toxicity and GV1001 pharmacodynamics. The dosing regimen and route of administration used in mice may not fully replicate the pharmacokinetic profiles observed in clinical settings. The tumor microenvironment and systemic immune interactions in murine models are less complex than those in human cancers, potentially affecting the assessment of GV1001’s dual effects on tumor suppression and nephroprotection. Despite these limitations, the mouse model remains a valuable preclinical tool for evaluating mechanistic pathways and therapeutic potential before human translation. Further studies, including pharmacokinetic, pharmacodynamic analyses and organoid models will be essential to confirm GV1001’s safety and efficacy in humans. Ultimately, these preclinical findings should lead to clinical trials evaluating GV1001 to improve the safety and tolerability of cisplatin-based cancer treatments and chemotherapy-induced nephrotoxicity.

## 5. Conclusions

GV1001 protects against cisplatin-induced nephrotoxicity by suppressing tubular damage, inflammation, EMT, and apoptosis, while preserving mitochondrial integrity and ATP production in renal epithelial cells. By restoring ETC function through likely binding cardiolipin, GV1001 interrupts the cascade of cisplatin-induced mitochondrial dysfunction that drives kidney injury. These findings position GV1001 as a promising mitochondria-targeted therapy to reduce cisplatin-associated nephrotoxicity.

## Data Availability

Data is available upon request to the corresponding authors.

## Supplementary Materials

The following supporting information can be downloaded at: https://www.mdpi.com/article/10.3390/cells14221818/s1, Figure S1. GV1001 abrogated cisplatin-induced apoptosis in renal tissues; Figure S2. GV1001 alleviated cisplatin-induced cellular ROS levels in HK-2 cells; Figure S3. GV1001 alleviated cisplatin-induced mitochondrial dysfunction in NHRECs; Figure S4. GV1001 reversed the inhibitory effect of cisplatin on the expression of electron transport chain (ETC) complexes in HK-2 cells; Figure S5. GV1001 alleviated the inhibitory effect of cisplatin on the expression of electron transport chain (ETC) complexes in NHRECs; Figure S6. GV1001 alleviated cisplatin-induced apoptosis in HK-2 and NHRECs; Table S1. The primer sequences for quantitative reverse transcription–polymerase chain reaction (RT-qPCR).

## Abbreviations

The following abbreviations are used in this manuscript:

CIN	Cisplatin-Induced Nephrotoxicity
FBS	Fetal Bovine Serum
PBS	Phosphate-Buffered Saline (PBS)
H&E	Hematoxylin and Eosin
PARP	Poly (ADP-Ribose) Polymerase
γ-H2AX	Gamma-H2A histone family member X
DAPI	4′,6-diamidino-2-phenylindole
cDNA	Complementary DNA
GAPDH	Glyceraldehyde-3-phosphate dehydrogenase
DMSO	Dimethyl Sulfoxide
hTERT	Human Telomerase Reverse Transcriptase
KIM-1	Kidney Injury Molecule-1
NGAL	Neutrophil Gelatinase-Associated Lipocalin
TUNEL	Terminal Deoxynucleotidyl Transferase dUTP Nick-End Labeling
IF	Immunofluorescence
ELISA	Enzyme-Linked Immunosorbent Assay
IL-6	Interleukin-6
IL-1β	Interleukin-1β
TNF-α	Tumor Necrosis Factor-alpha
MMP	Mitochondrial Membrane Potential
FITC	Fluorescein Isothiocyanate
BUN	Blood Urea Nitrogen
α-SMA	Alpha Smooth Muscle Actin
EMT	Epithelial-to-Mesenchymal Transition
Oct-02	Organic Cation Transporter 2
NHREC	Normal Human Renal Epithelial Cell
ETC	Electron Transport Chain
COX-4	Cytochrome c Oxidase Subunit IV
PI(4,5)P_2_	Phosphatidylinositol 4,5-Bisphosphate
NAO	10-N-nonyl Acridine Orange
NDUFS1	NADH Dehydrogenase (ubiquinone) Fe-S Protein 1
SDHB	Succinate Dehydrogenase Complex Iron–Sulfur Subunit
UQCRFS1	Ubiquinol–Cytochrome C Reductase Rieske Iron–Sulfur Polypeptide 1
p-p65	Phosphorylated NF-kB p65
ROS	Reactive Oxygen Species
MitoSox	Mitochondrial Superoxide Indicator
CMSRos	Chromomethy-X-Rosamine
dUTP	deoxyuridine triphosphate
RT-qPCR	Reverse Transcription quantitative polymerase Chain Reaction
DMEM	Dulbecco’s Modified Eagle Medium
HEPES	4-(2-Hydroxuethyl)-1-piperazineethanesulfonic acid
