# Supplementary material for: GV1001, an hTERT-Derived Peptide, Prevents Cisplatin-Induced Nephrotoxicity by Preserving Mitochondrial Function
Source: Cells. 2025 Nov 19;14(22):1818. doi: 10.3390/cells14221818 (PMC12651939; doi:10.3390/cells14221818)
Supplement: Supplementary file 1 [file cells-14-01818-s001.zip › cells-3899928-supplementary.pdf]

Supplementary data:

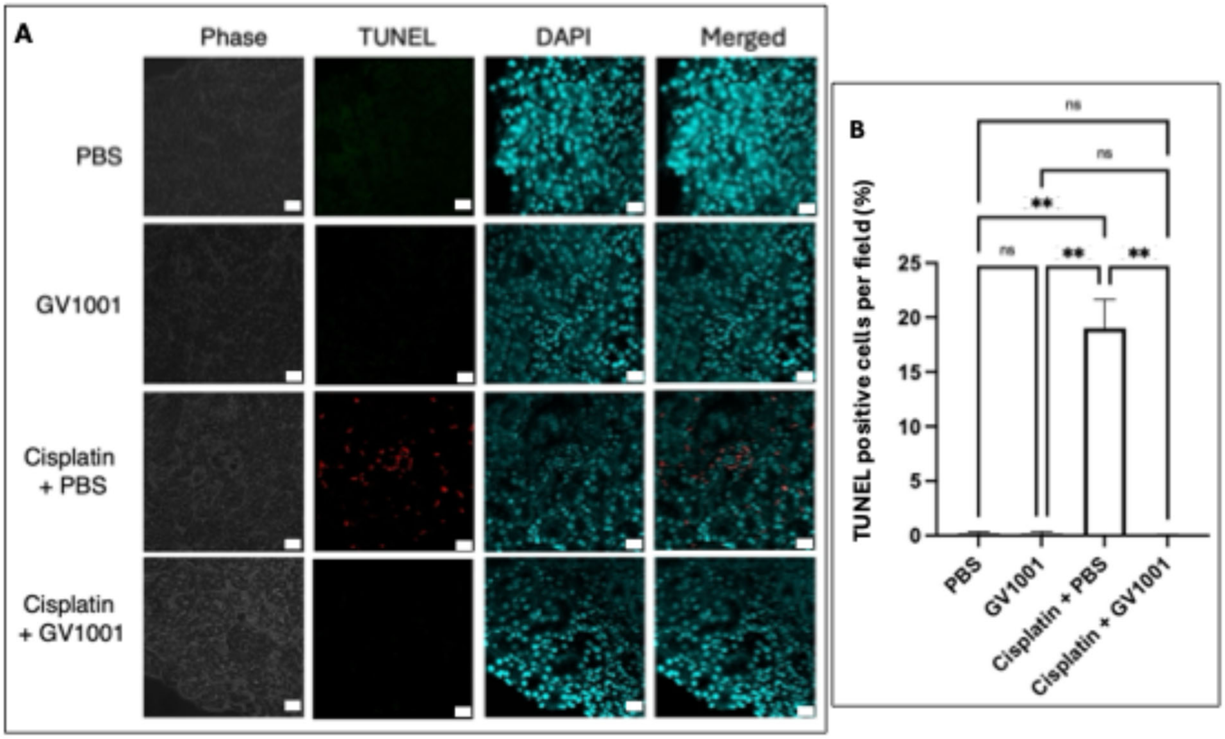

Figure S1. GV1001 abrogated the cisplatin-induced apoptosis in renal tissues. **A.** Representative immunofluorescent TUNEL staining in renal tubular epithelium from mice treated with PBS, GV1001, cisplatin alone or in combination with GV1001. TUNEL-positive cells were in red and DAPI counterstaining nucleus was in blue. Scale bar: 20 μm. **B.** Quantification of TUNEL-positive cells with ImageJ software (mean ± SEM). Data were analyzed by one-way ANOVA. ns, not significantly different; $^*P < 0.05$; $^{**}P < 0.01$ with n = 8 per group. All experiments were performed in quintuplicate.

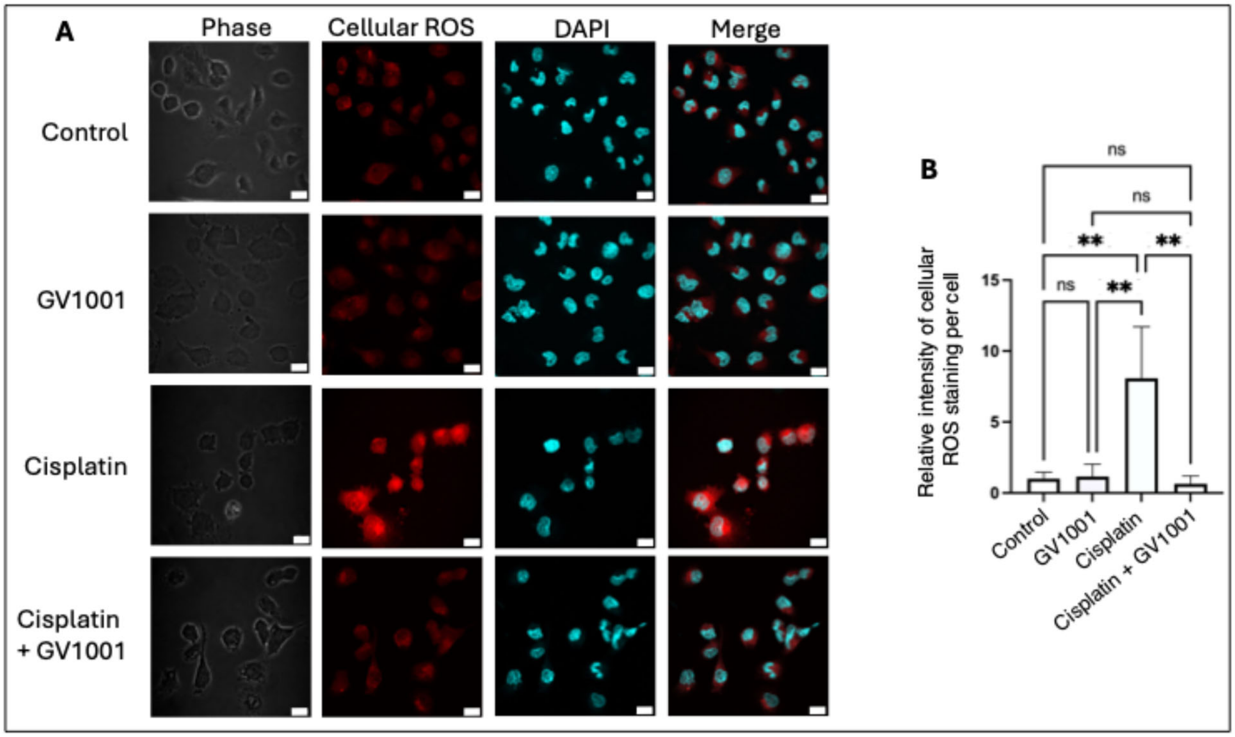

Figure S2. GV1001 alleviated the cisplatin-induced cellular ROS levels in HK-2 cells. **A**. Representative immunofluorescence images of cellular ROS detected by fluorometric intracellular ROS probe (red) in HK-2 cells treated with cisplatin, GV1001, or the combined treatment of cisplatin and GV1001. Scale bar: 20 μm. **B.** Quantification of the intensity of cellular ROS per cells using ImageJ analysis. Data were analyzed by one-way ANOVA; ns = not significantly different, *P < 0.05, **P < 0.01. All experiments were performed in quintuplicate.

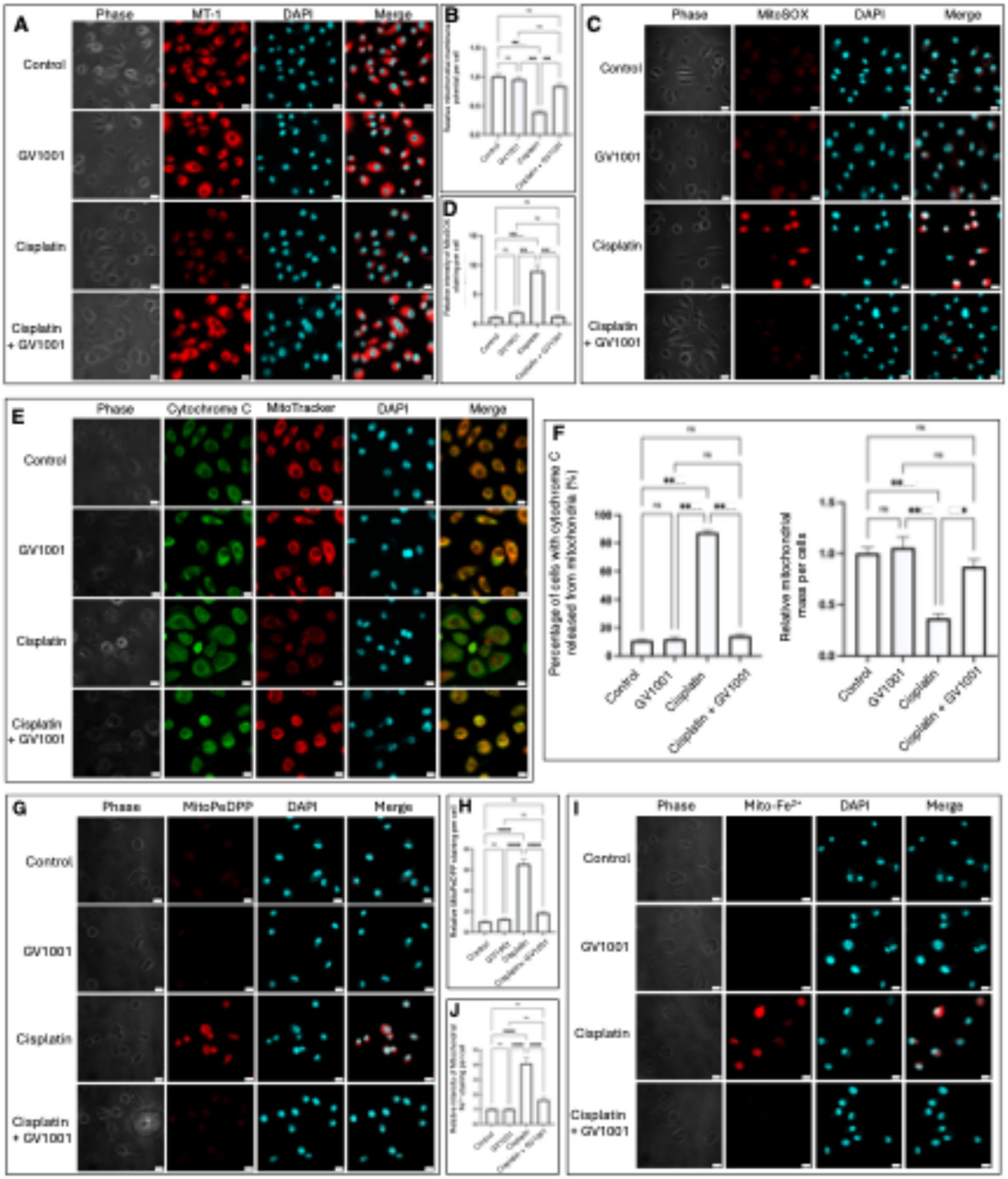

Figure S3. GV1001 alleviated cisplatin-induced mitochondrial dysfunction in NHREC cells. **A**. Representative immunofluorescence images of mitochondrial membrane potential (MMP) detected by MT-1 (red) in NHREC cells treated with cisplatin, GV1001, or a combination of cisplatin and GV1001 for 24 hr. Scale bar: 20 μm. **B.** Quantification of MMP using ImageJ analysis. **C.** Representative immunofluorescence images of mitochondrial ROS (Red) in NHREC cells treated with cisplatin, GV1001, or a combined treatment of cisplatin and GV1001 for 24 hr. Scale bar: 20 μm. **D.** Quantification of mitochondrial ROS using ImageJ software. Representative immunofluorescence staining of cytochrome c

(green) and mitochondrial mass detected by MitoTracker Red CMXRos (red) **(E)** and lipid peroxidation (MitoPeDPP staining) (**G**) mitochondrial ferrous iron (Mito-Fe^{2+}) **(I)** in NHREC exposed to GV1001 alone or together with cisplatin. Nuclei were counterstained with DAPI (blue). Scale bar: 20 μm. Quantification of cells with cytosolic cytochrome c per field and mitochondrial mass **(F)**, mitochondrial lipid peroxidation **(H)** and mitochondrial ferrous iron **(J)**, using ImageJ analysis. Data were analyzed by one-way ANOVA; ns = not significantly different, * P<0.05, ** P<0.01, **** P<0.0001. All experiments were conducted in quintuplicate.

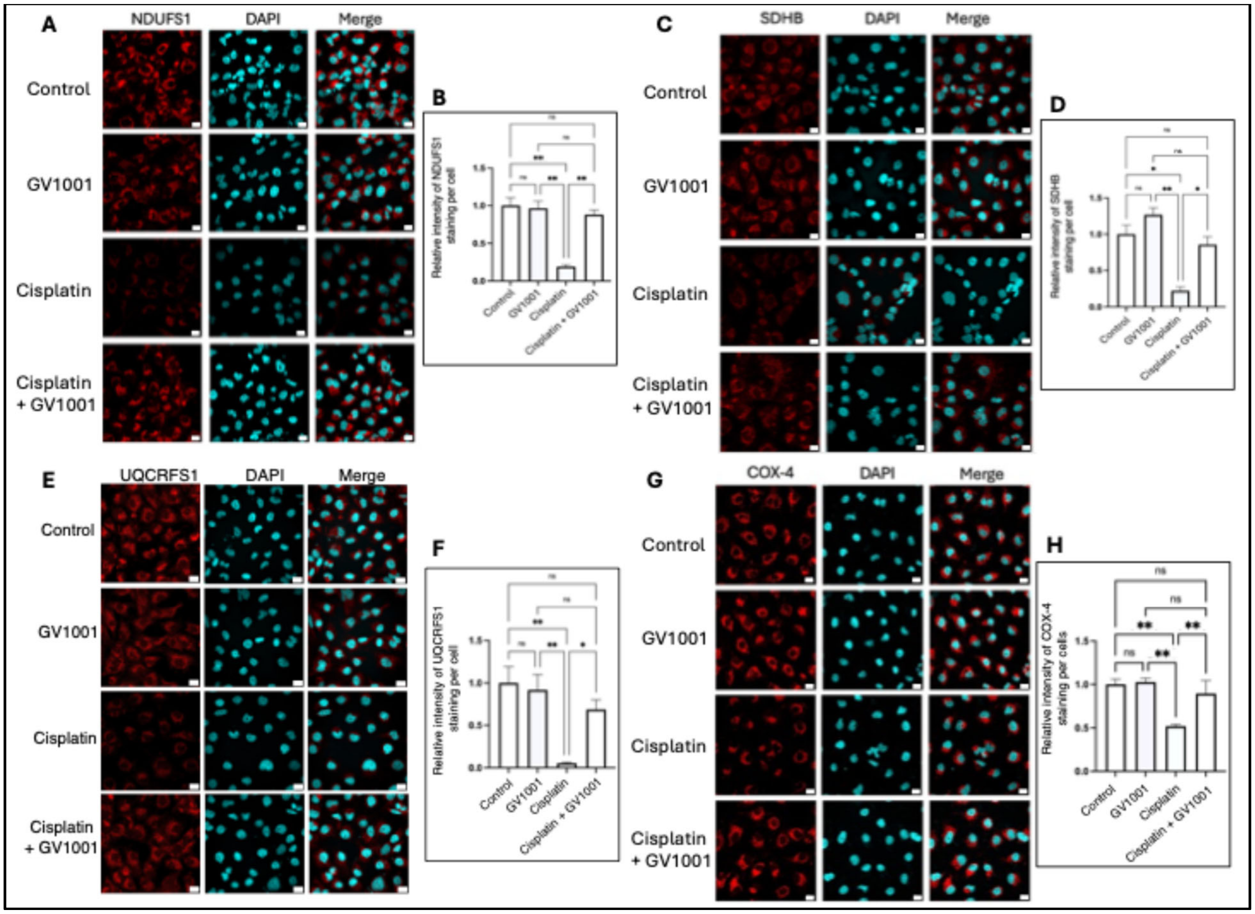

Figure S4. GV1001 reversed the inhibitory effect of cisplatin on the expression of electron transport chain (ETC) complexes in HK-2 cells. **A. C. E. G.** Representative immunofluorescent staining images of NDUFS1 (complex I), SDHB (complex II), UQCRFS1 (complex III) and COX-4 (complex IV) in HK-2 cells exposed to cisplatin, GV1001 alone or together with cisplatin for 24 hrs. ETC complex proteins were stained in red color. Nuclei were counterstained with DAPI (blue). **B. D. F. H.** Quantification of ETC complex expression levels using ImageJ analysis. Results were presented as means ± SEM and analyzed by one-way ANOVA; ns, not significantly different; * P<0.05; ** P<0.01. Scale bar: 20 μm. All experiments were performed in quintuplicate.

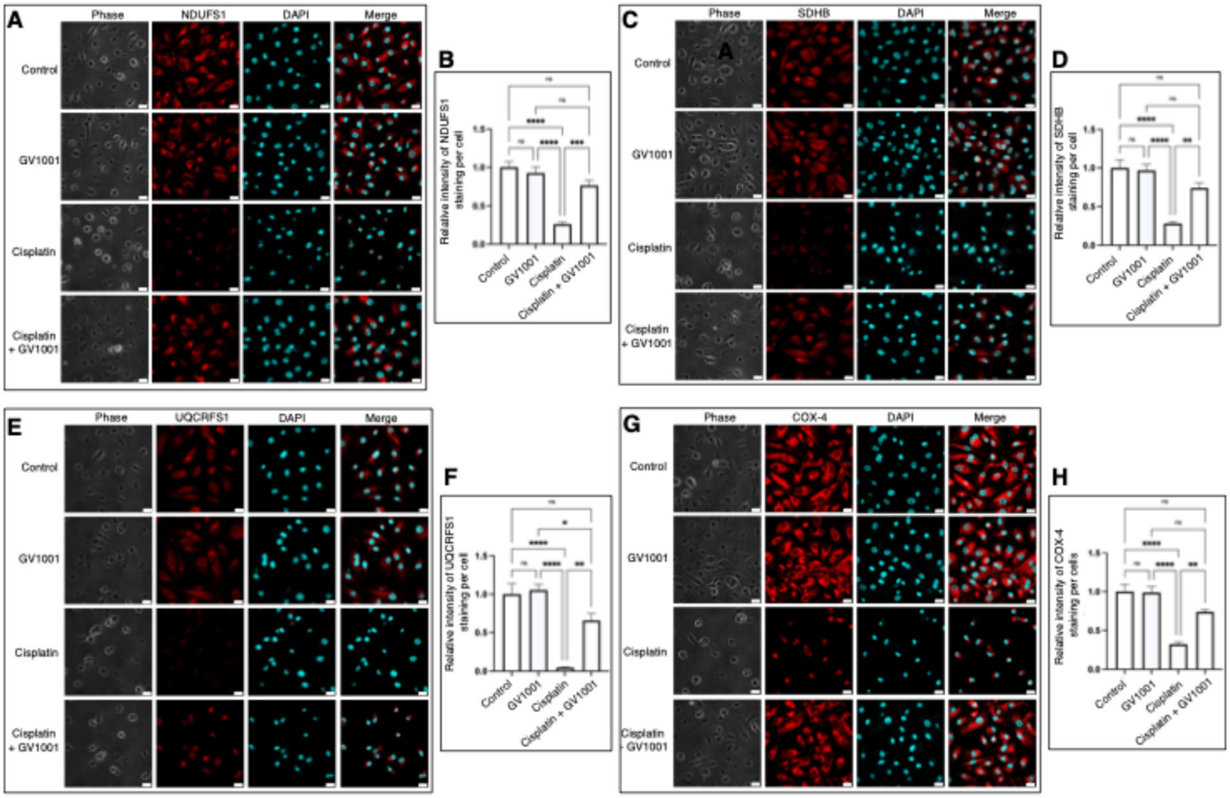

Figure S5. GV1001 alleviated the inhibitory effect of cisplatin on the expression of electron transport chain (ETC) complexes in NHREC. **A. C. E. G.** Representative immunofluorescent staining images of NDUFS1 (complex I), SDHB (complex II), UQCRFS1 (complex III) and COX-4 (complex IV) in NHREC cells exposed to cisplatin, GV1001 alone or together with cisplatin for 24 hrs. ETC complex proteins were stained in red. Nuclei were counterstained with DAPI (blue). **B. D. F. H.** Quantification of ETC complex expression levels using ImageJ analysis. Results were presented as means ± SEM. Data were analyzed by one-way ANOVA; ns, not significantly different; * $P<0.05$; ** $P<0.01$ and **** $P<0.0001$. Scale bar: 20 µm. All experiments were performed in quintuplicate.

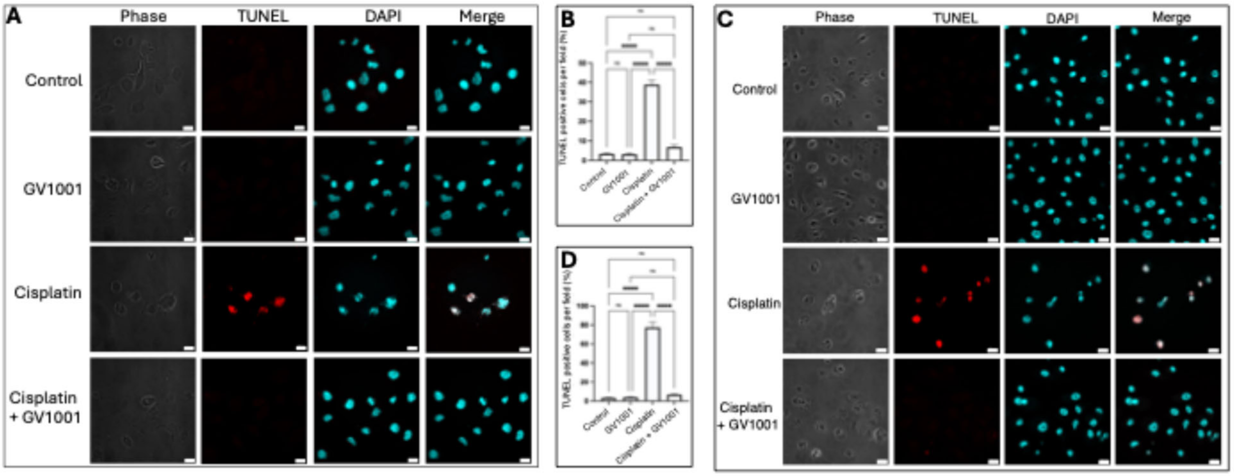

Figure S6. GV1001 alleviated cisplatin-induced apoptosis in HK-2 and NHREC cells. **A, C.** Representative immunofluorescent TUNEL staining in HK-2 **(A)** and NHREC cells **(C)** treated with GV1001, cisplatin alone or in combination with GV1001. TUNEL-positive cells were stained in red, and nucleus was counterstained with DAPI (blue). Scale bar: 20 μm. **B, D.** Quantification of TUNEL-positive cells in HK-2 **(B)** and NHREC cells **(D)** exposed to GV1001, cisplatin alone or in combination with GV1001 with ImageJ software. Results were presented as means ± SEM. Data were analyzed by one-way ANOVA. ns, not significantly different; **** $P<0.0001$. All experiments were performed in quintuplicate.

Supplemental Table:

Table S1. Sequences of the primers for quantitative reverse transcription-polymerase chain reaction (RT-qPCR).

Genes	Forward primer 5′-3′	Reverse primer 5′-3′
mIL-1β	CACAGCAGCACATCAACAAG	GTGCTCATGTCCTCATCCTG
mTNF-α	TCAGGTTGCCTCTGTCTCAG	GCTCTGTGAGGAAGGCTGTG
mIL-6	TGGGACTGATGCTGGTGACA	GCCTCCGACTTGTGAAGTGGT
mGAPDH	AGCTTGTCATCAACGGGAAG	TTTGATGTTAGTGGGGTCTCG
mKIM-1	CTGGAATGGCACTGTGACATCC	GCAGATGCCAACATAGAAGCCC
mNGAL	ATGTCACCTCCATCCTGGTCAG	GCCACTTGCACATTGTAGCTCTG
